# A Scoping Review on Technological Literacy in Biomedical Laboratory Science and Implications for Educational Development

**DOI:** 10.1007/s40670-026-02761-0

**Published:** 2026-06-09

**Authors:** Filis Cramer Necip, Henriette Lorenzen, Marianne Ellegaard

**Affiliations:** 1 Department of Pathology, Copenhagen University Hospital (Rigshospitalet), Copenhagen, Denmark; 2 Faculty of Health, University College Copenhagen, Copenhagen, Denmark

**Keywords:** Technological literacy, Health education, Artificial intelligence, Computational thinking, Data literacy

## Abstract

Technological literacy (TL) is increasingly critical in biomedical laboratory science (BLS). This scoping review examines how TL in conceptualized in BLS, drawing on Orlikowski & Gash´s socio-technical framework and the DigComp 2.2 digital competence framework. Nineteen sources were analyzed, revealing a dichotomy between backward-looking perspectives, emphasizing operational proficiency and routine technology use, and forward-looking perspectives, highlighting AI, computational thinking, data literacy, and strategic engagement with technology. These findings call for BLS education to support foundational digital skills, critical reflection, and problem-solving to prepare students for the evolving professional technological landscape.

## Introduction

In a healthcare landscape characterized by rapid technological innovation and increasing complexity, digital health—spanning technologies like big data analytics, artificial intelligence, and robotics—is playing an increasingly critical role in improving health outcomes [1]). This is particularly significant in biomedical laboratory science (BLS), where technology is integral to practice, and drives advances in diagnostics, automation, data analysis, and personalized medicine. For example, in digital pathology, BLS professionals are increasingly handling slide scanning, quality assurance of digital images, and even contributing to image analysis using AI-assisted tools [2].

These developments are transforming the role of BLS professionals, who are increasingly expected to contribute not only to routine testing but also to complex clinical decision-making [3–5]. This complexity is further amplified by the fact that the BLS profession spans a broad range of clinical subspecialties and technological contexts. All this calls for BLS education to play a central role in addressing these changing demands on the healthcare sector by equipping future professionals with the competencies needed to navigate advanced laboratory technologies. The question is: What does this entail, i.e. what is needed to equip BLS for the future of technology? To answer this question, it is central to identify the underlying competencies that enable BLS to engage critically and effectively with technology. A way to understand the intersection between professional practice and technological capability is through the concept of technological literacy (TL).

To the best of our knowledge, no previous review has systematically explored technological literacy (TL)— seen as the ability to use, manage, assess and understand technology [6] within BLS. The aim of this review is therefore to assess published studies and resources on professional competencies that address aspects of TL in the BLS profession. Insights gained from this review have the potential to inform educational practices, guide curriculum development, and shape policy decisions, ensuring that BLS education continues to align with the needs of a rapidly evolving healthcare environment.

Accordingly, this scoping review seeks to discuss: What does TL encompass for BLS? Is there consensus in the literature, and if not, what are the areas of divergence? What does this imply for teaching and training in BLS education, to prepare students for technological and digital healthcare?

## The Professional Scope of Biomedical Laboratory Science and Implications for Technological Literacy

BLS is a relatively new profession, with varying international titles for professionals responsible for laboratory diagnostics and research in health care. While the authorized term is BLS, titles such as biomedical scientists, medical laboratory scientists, or bioanalysts are also widely used [7]. The field spans a range of clinical subspecialities, including clinical chemistry, haematology, immunology, microbiology, histopathology, molecular diagnostics, and transfusion medicine, each characterized by distinct technological infrastructures, procedural standards, and professional cultures [7]. BLS professionals perform, validate, and interpret laboratory analyses, encompassing specimen collection and processing, operation of diagnostic instruments, implementation of quality assurance procedures, and communication of results to clinicians, that support clinical decision-making, patient monitoring, and therapeutic interventions [7]. According to the International Federation of Biomedical Laboratory Science (IFBLS) [7], BLS professionals are expected to demonstrate competencies spanning specimen management and analysis, laboratory informatics, quality assurance, and patient-centered communication. These core competencies are grounded in an understanding of both scientific principles and technical processes and evolve in tandem with advances in laboratory technology. We therefore consider it important to clarify what TL entails for BLS.

## Methodology

Our analysis of the literature draws on two complementary theoretical frameworks: Orlikowski & Gash´s socio-technical perspective [8], and the European Commission’s DigComp 2.2 framework [9]. This approach was chosen because together these two frameworks combine theoretical abstract conceptualizations of technology [8] with specific measurable dimensions of digital competencies [9]. Orlikowski and Gash´s framework [8] was originally developed to understand how technologies are perceived by different stakeholders in a workplace setting [8]. This framework can serve as a qualitative lens to examine how various stakeholders in workplace training of BLS, perceive and interpret TL. Giving a different perspective, the DigComp framework [9] provides a structured, quantitative approach to analyzing for specific digital competencies. By combining these perspectives, we can both examine *how* TL in BLS is understood, *which* technological competencies are seen as central and evaluate the *degree* to which specific digital competencies are seen as embedded in BLS practice. The combined use of the two frameworks thus enhance our ability to understand how TL is conceptualized and operationalized within BLS.

In the following sections, we describe how these frameworks were methodologically employed to classify and interpret the included sources.

### Technological Frame by Orlikowski & Gash

**Fig. 1:**
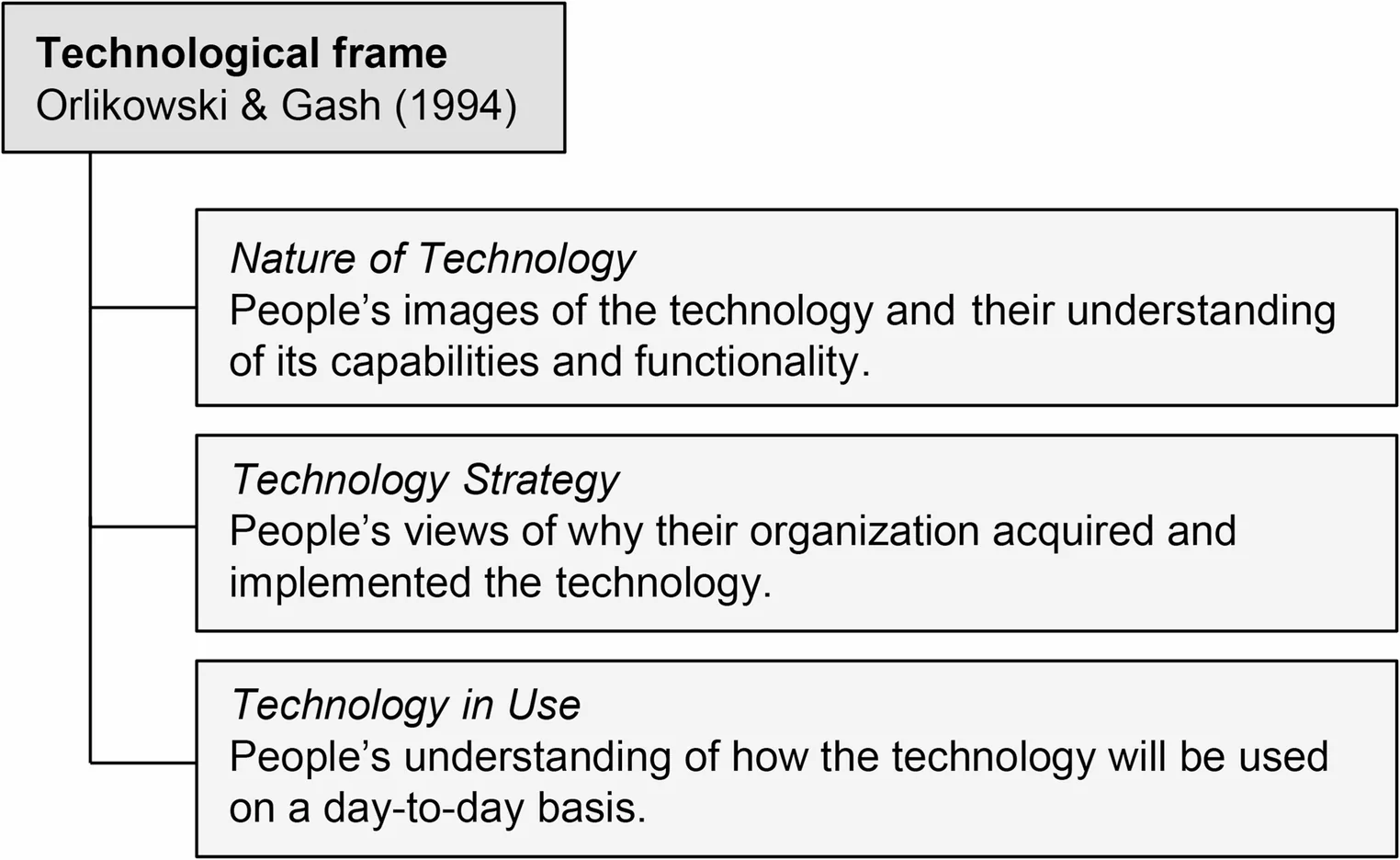
Domains of the technological frame by Orlikowski & Gash [8]

To guide the analysis of *how* TL is understood and applied in BLS, we employed the Orlikowski & Gash framework [8], which divides the understanding of technological development into three interrelated domains (see Fig. 1). Together, these domains offer a comprehensive perspective on how technology is understood, implemented, and applied in BLS practice.

The *Nature of Technology* domain concerns understanding the intrinsic properties and design principles of technological systems, including their capabilities and functionality [8]. In the context of BLS, this could involve understanding the fundamental mechanics behind diagnostic technologies or data acquisition.

The second domain, *Technology Strategy*, focuses on the broader planning and integration of technology within organizations, including the rationale and expected benefits [8]. In laboratory environments, this could include decisions such as choosing technology to enhance diagnostic accuracy, streamline workflow processes or meet regulatory standards.

The third domain, *Technology in Use*, relates to the understanding of how the technology will be used and required competencies for effective operation [8]. In a BLS context, this could refer to all aspects of operating laboratory technology, such as maintenance, calibration, quality control, and managing technical challenges.

### The Digital Competence Framework for citizens (DigComp): Structure and Dimensions

**Fig. 2:**
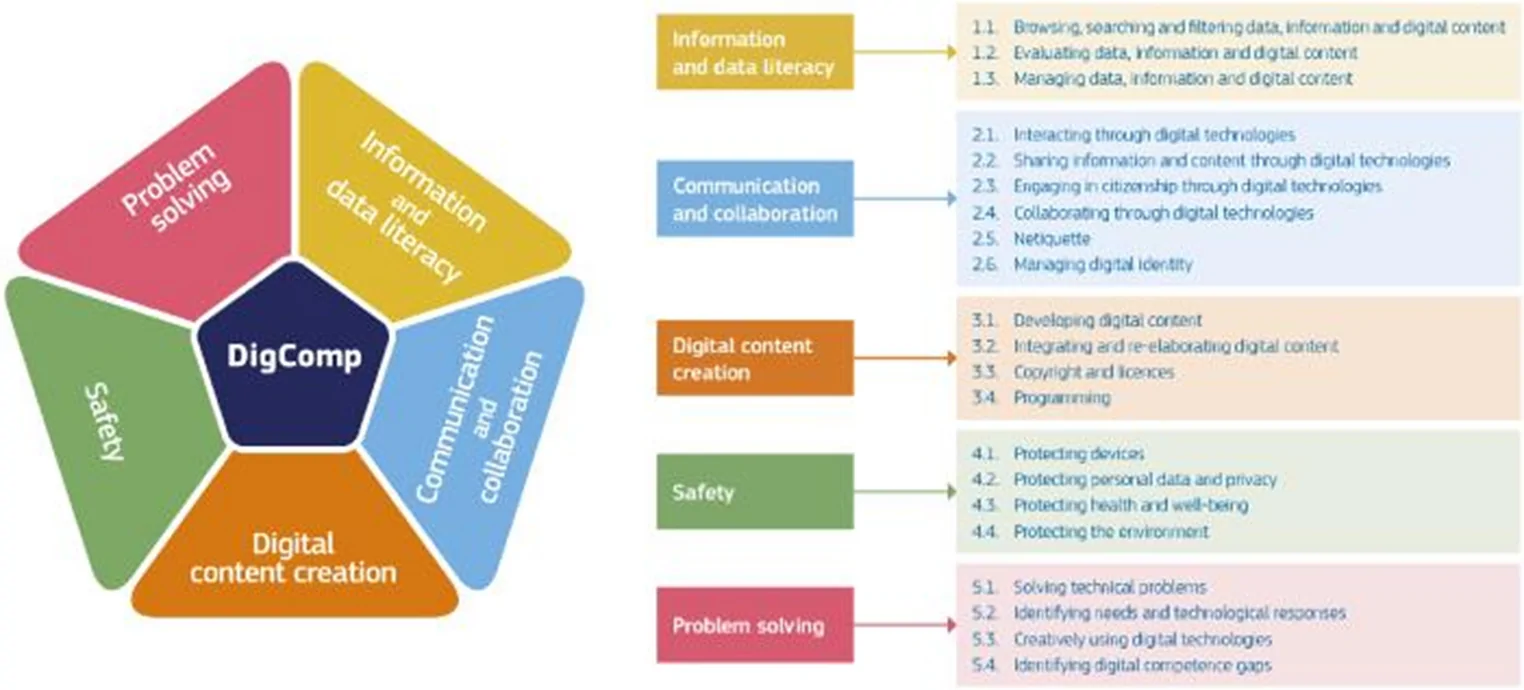
The digital competence framework for citizens (DigComp) outlines five main dimensions, each comprising sub-competences that together provide a comprehensive structure for assessing and developing digital skills [9]

To guide the analysis of the *degree* to which specific digital competencies are represented and/or emphasized within the reviewed literature, we employed the European Commission’s DigComp 2.2 [9]. Although originally developed to address citizens’ digital skills, the DigComp offers a comprehensive and adaptable model that can be applied in a professional context for identifying digital competencies. The framework comprises five dimensions, each subdivided into specific sub-competences, resulting in a total of twenty-one elements that collectively encompass the breadth of digital competencies [9] (see Fig. 2).

As illustrated in Fig. 2, the five dimensions are arranged in a pentagonal structure, reflecting their interdependence and equal significance. The dimension of *Information and Data* emphasizes the ability to locate, evaluate, and manage digital content, including critically assessing its reliability and organizing it effectively. The dimension of *Communication and Collaboration* focuses on digital interaction across contexts, including sharing information, collaborating through digital means, and managing digital identity. *Digital Content Creation* addresses the ability to produce and adapt digital materials, as well as understanding copyright and fundamental programming. The *Safety* dimension focuses on data protection, digital well-being, and awareness of environmental impacts associated with digital practices. Finally, the dimension of *Problem Solving* involves the capacity to identify technical issues, select digital solutions, apply technologies creatively, and engage in ongoing self-assessment of one’s digital development [9].

In the context of BLS, DigComp enables a systematic approach to quantifying the digital dimensions of TL, thereby complementing the qualitative analysis by Orlikowski & Gash´s framework [8].

### Review Design and Reporting Standards

This scoping review was conducted systematically and reported according to the Preferred Reporting Items for Systematic Reviews and Meta-Analyses extension for Scoping Reviews (PRISMA-ScR) checklist [10].

The research questions were formulated using the Population, Exposure, Comparator, and Outcomes (PECO) framework [11]. Due to the diversity in the terminology used about the BLS profession, we included a wide range of international professional titles in our search strategy —such as Biomedical Laboratory Scientist, Medical Engineers, Medical Scientist, Medical Laboratory Technologist, Medical Laboratory Scientist, Biomedical Scientist, Biomedicinska analytiker, Medical/Clinical Laboratory Scientist and Pathology Laboratory Technicians, Pathologists’ assistants, Bioanalysts as well as clinical subspecialties in microbiology, pathology, molecular genetics, immunology, hematology, physiology, and nuclear medicine.

### Search Strategy and Data Sources

An initial search was conducted in Medline and the Cochrane Library to check whether existing reviews had addressed this specific area. The search strategy was developed iteratively. Initial searches were conducted independently by two authors, after which results were compared and discussed within the full author group. Based on these discussions, search terms and scope were refined, and additional searches were conducted to ensure both breadth and relevance of the included sources. The final comprehensive search query was constructed using Boolean operators, truncation (*), and comprehensive search terms that encapsulated the thematic aspects of technological literacy in BLS practice and education [12, 13]. Additional databases and registers including Google Scholar, PubMed, ERIC, EMBASE and Web of Science were searched. To identify national standards and guidelines for competencies, we consulted the register lists of the IFBLS, which provided access to international member organizations in the field of BLS. Finally, a snowballing technique, screening reference lists, citations, and conference contributions, was applied to ensure the search’s comprehensiveness.

**Fig. 3:**
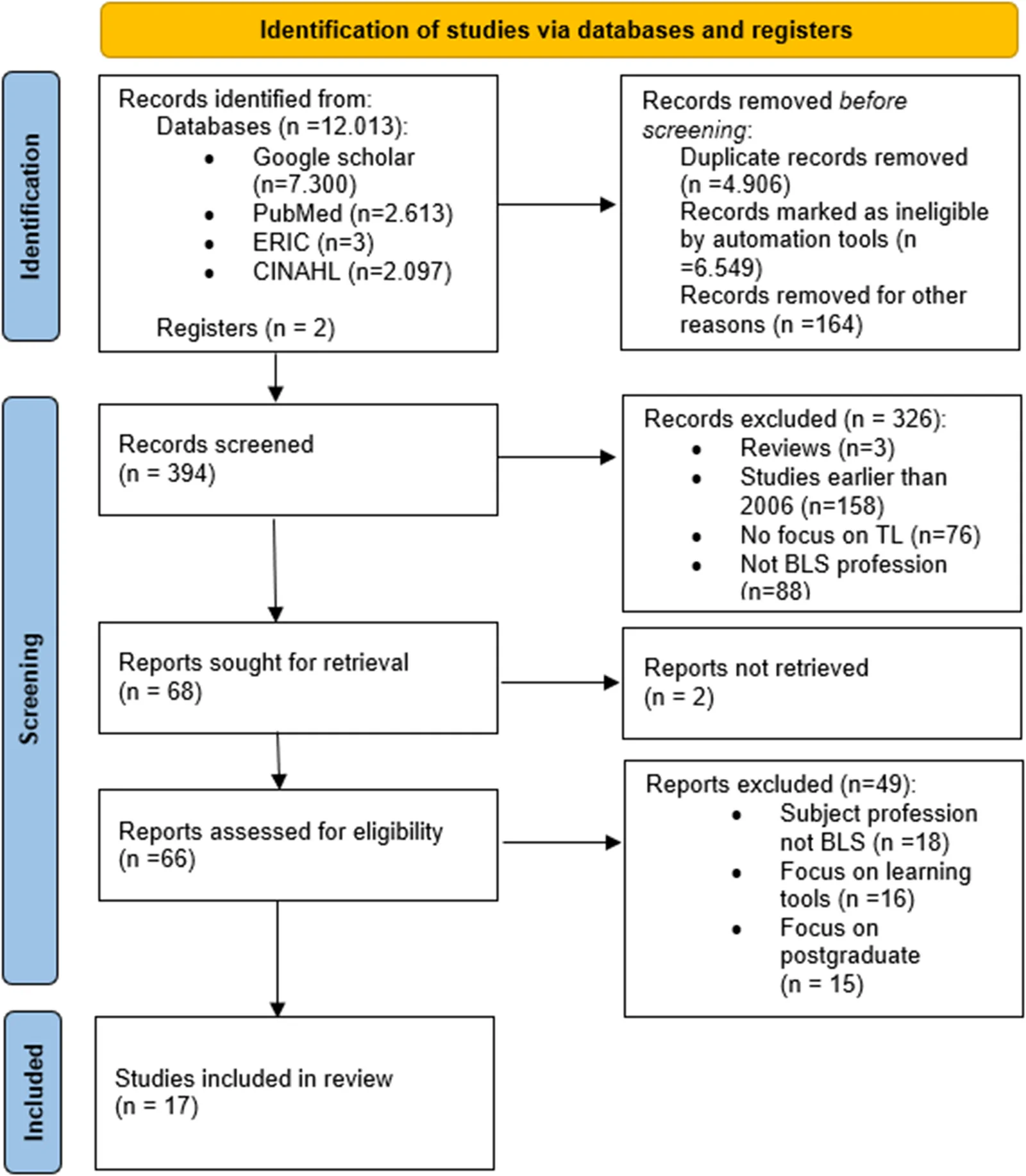
Summary of the identification, screening, and selection process of studies included in the review using PRISMA flow chart [12]

To ensure a systematic and transparent selection process, inclusion and exclusion criteria were defined prior to inclusion (see Fig. 3).

Studies were included if they:

- addressed biomedical laboratory science (BLS) or closely related laboratory professions terminology.
- focused on technological literacy, digital competence, or technology-related competencies.
- were relevant to education, professional practice, competency frameworks, or policy documents.
- were published in English or Scandinavian languages.

Studies were excluded if they:

- did not relate to BLS or comparable laboratory professions.
- did not address technological or digital competencies.
- focused exclusively on digital learning tools, postgraduate or professional development.
- were published before 2006, to ensure relevance to contemporary technological developments.

### Data Extraction and Classification

The included sources in this review span a wide variety of publication types, including empirical research articles, opinion pieces, as well as non-peer-reviewed international or institutional competency standards and guidelines, and strategic reports. Although not peer-reviewed, the standards, guidelines and reports are included for their practice-relevant insights and their role in shaping professional expectations regarding TL.

In the initial phase of data extraction, we categorized each source according to publication type, intended audience, and context of production (e.g., policy, practice, education, research).

We adopted an iterative, interpretive approach to classification and analysis. All three authors independently reviewed the full set of included sources and conducted preliminary assessments of their relevance to both analytical frameworks. Two authors extracted relevant excerpts. Across both frameworks, one author categorized the extracted relevant excerpts, and the categorization was subsequently discussed by all authors to reach consensus. This process was deliberately dialogic and reflexive, allowing the research team to negotiate interpretations, resolve ambiguities, and reach consensus on how each source should be categorized and analyzed. Each source was examined in relation to Orlikowski and Gash’s framework [8], while a subset, selected because they provided sufficient detail on digital practices to allow systematic and competence-based assessment, was further analyzed using the DigComp 2.2 framework [9].

To systematically assess the extent to which the five DigComp dimensions are reflected in each of the selected sources, we applied a structured quantitative scoring procedure across the 21 subcategories comprising the framework´s five dimensions [9] using a 5-point Likert-scale:

1. Not addressed at all
2. Slightly addressed
3. Moderately addressed
4. Well addressed
5. Fully integrated

This scoring was interpretative, relying on informed judgement regarding the presence, depth, and contextual relevance of each DigComp subcategory within each source. In particular, the scoring distinguished between implicit and explicit representations of competencies, where higher scores required clearly articulated and well-developed descriptions of competencies within the source. To ensure diverse perspectives and to minimize individual bias, all authors conducted their assessment independently.

After independent scoring, results were compared and discussed. In the few cases of major discrepancies, defined as a difference of two or more points between authors, scores were discussed in detail until consensus was reached. For example, discrepancies arose when competencies were described implicitly rather than explicitly, leading to differences in how authors interpreted the depth of representation within a given DigComp dimension. Minor discrepancies of one point were settled by adopting the majority score as the final rating. Figures were created using the official DigComp 2.2 online visualization tool provided by the European Commission [14].

## Results

### Descriptive Characteristics of the Included Sources

This scoping review identified 17 relevant sources on BLS technological and digital competences (Fig. 3). Our analysis revealed two distinct perspectives in the sources; a backward-looking perspective on professional competences, and a forward-looking perspective. Thirteen sources describe existing BLS competences representing the backward-looking perspective [7, 15–26], while four studies adopted a forward-looking perspective exploring competencies needed for future BLS practice [2, 27–29].

The sources with a backward-looking perspective mainly comprised standards and guidelines on core competencies defined by professional organizations [7, 15, 16, 18, 24–26], as well as studies on teaching and learning activities [19, 23], studies on educational syllabi [21, 22], and Delphi studies on competency profiles of novice BLS professionals [17, 20]. The forward-looking sources included qualitative studies on recommended competencies of BLS [2, 27], an opinion paper [29], and a review on recommended competencies [28].

Most of the sources in both above groups covered a broad spectrum of clinical subspecialties, with a few exceptions that focused on a specific clinical specialty: two on pathology [2, 23] and one on medical imaging [15].

The majority of sources were European, with a few from North America, Australia, East Asia and the Middle East. Additionally, one source represented a global BLS organization. No sources were identified from South America or Central and South Asia.

### Approaches and Attitudes to Technological Literacy Using the Orlikowski & Gash Framework on Sources with a Backwards-Looking Perspective

This group of sources includes six studies [17, 19–23] and seven sets of guidelines/standards on BLS competences [7, 15, 16, 18, 24–26] which were analyzed based on Orlikowski & Gash´s domains [8] to examine what, within each of these domains, are identified as core competencies and how they are described.

Studies and guidelines addressing themes within the *nature of technology* reveal both broad and specific competencies. A competency, which is often highlighted, is the importance of understanding the fundamental principles of common technologies to ensure their effective use and application in clinical settings [7, 22, 24, 26]. Particular attention is given to core concepts related to the design and operation of laboratory instruments. Supporting this understanding allows students to move from basic equipment to more advanced technologies [7, 22]. Some sources focus on specific technologies, such as understanding the functionality of Laboratory Information Systems (LIS) [19], and the technical aspects of digital imaging, including workflow processes [23]. However, most of the included studies and guidelines do not address the *nature of technology* within their curriculum or as part of core competencies [15–18, 20, 21, 25].

The incorporation of *technology strategy* is addressed in the sources from a variety of perspectives. The importance of understanding the purpose of technology is emphasized; this can be, for example, ensuring traceability from patient identification to reporting, and restricting access to results to authorized individuals [18, 20]. Other sources highlight understanding the broader role of information systems in laboratory workflows [19], the significance of digital tools in statistical analyses and bioinformatics [21], adapting practice to new technologies [26] and optimizing processes to enhance efficiency and environmental sustainability [18]. Additionally, an integrated approach to technology in education is reported, stressing adaptability and continuous learning [7, 22], along with the involvement of staff in training both students and colleagues on the operation of equipment [7, 18]. Furthermore, the value of understanding the benefits of integrating knowledge across disciplines, such as technology, medicine, physical sciences, and statistics, to improve patient care is emphasized [7]. However, several sources do not explicitly address technology strategy as part of their curriculum or core competencies [15–17, 23–25].

Finally, with regard to *technology in use*, most sources emphasize, as important, proficiency in operating and maintaining various laboratory and diagnostic equipment, covering areas such as calibration, quality control, process monitoring and troubleshooting [7, 16, 18, 20, 22, 24–26]. Some sources also focus on specific laboratory and diagnostic technologies, including ultrasound, and imaging equipment [15], ECG examinations [20], point-of-care testing and telemedicine applications [22] and the use of digital pathology systems, including image acquisition and algorithm-based analysis [23]. Moreover, digital skills are highlighted, including data management [7, 16] and Information Communication Technological (ICT) proficiency, data literacy, and scientific communication [21]. The operation of LIS for patient sample documentation, result reviews, and communicating results is also emphasized [18, 19, 24]. Only one source does not explicitly mention competencies related to technology use, although these are assumed relevant to several of the laboratory skills reported [17].

Overall, this analysis reveals that while most of the included sources emphasize *technology in use*, fewer address the *nature of technology* or *technology strategy* within current competences of BLS. The focus is primarily on practical competencies, such as operating laboratory equipment, digital skills, and LIS, with less attention given to the purpose of technology or its strategic role in professional practice. In addition, some studies only implicitly address technology.

Since we performed the analysis, new studies from Africa and South Asia, adopting a backward-looking perspective, have confirmed the strong emphasis on *technology in use*, with a prioritization of practical competencies in operating laboratory equipment [30, 31]. However, limited access to technology remains a critical issue in some countries [30]. Regarding the *nature of technology*, one of the sources highlights that beyond operational skills, it is essential to understand the underlying principles of the technologies used [31].

### Forward-Looking Competencies in Technological Literacy Using the Orlikowski & Gash Framework

We have categorized four studies [2, 27–29] as having a forward-looking perspective on TL in BLS and analyzed these based on Orlikowski & Gash´s domains [8] to outline competencies that should be integrated in future BLS.

Within the *nature of technology*, the need for a fundamental understanding of technology in laboratory medicine is emphasized, particularly in relation to artificial intelligence (AI) and computational skills. AI and computational thinking are identified as crucial competencies, requiring BLS professionals to build a strong foundation in areas such as AI algorithms, data literacy, and computational thinking [27, 28], and to understand the application of AI in healthcare for advancing laboratory practices [28]. This is further supported by an emphasis on developing technological understanding to advance BLS in digital pathology, particularly in areas such as whole slide imaging, digitally assisted image analysis, and AI applications with suggestions that BLS can have responsibility for the answer on simple diagnostic samples [2]. Furthermore, data management is highlighted, stressing the importance of understanding data structure, quality, and management to support AI-driven and digital workflows in laboratory medicine [2, 27–29].

None of the four studies explicitly discuss competencies within *technology strategy*. However, we detect aspects that point to the need for understanding the purpose behind the use of technologies. The importance of fostering an understanding of the purpose of social skills to support effective collaboration in decentralized, asynchronous work environments is emphasized [27]. The role of robot technology and digital pathology in quality assurance is highlighted, suggesting that understanding its purpose is essential for BLS [2]. The need to understand how AI can contribute to error reduction and test optimization in laboratory settings is stressed [28]. Additionally, the importance of empowering BLS professionals with an understanding of the skills needed to work with new instruments and software is underscored, to ensure a successful digital transformation in laboratory medicine [29].

Finally, regarding *technology in use*, the studies outline specific competencies required for BLS, emphasizing the importance of expertise in working with new instruments and software [29] and the need for digital skills such as big data analysis, basic programming, and machine learning [27, 28]. One source focuses on the technical aspects of data analysis and machine learning model training [27], while another highlights integrating AI, machine learning, and data analytics, to enhance result interpretation [28]. Additionally, digital pathology is emphasized, with a focus on the need for proficiency in scanning, calibration, IT troubleshooting, and the use of AI-assisted diagnostics [2].

Overall, this analysis reveals that the studies highlight the importance of both the *nature of technology* and *technology in use* in BLS programs, while *technology strategy* is more implicit. There is a clear focus on developing future competencies in digital skills, AI, and data analysis, alongside an increasing emphasis on understanding the purpose of technology, to enhance collaboration, optimize processes, and drive digital transformation in laboratory medicine.

### Analysis of Domains of Digital Competencies Using the DigComp 2.2 Framework

**Fig. 4:**
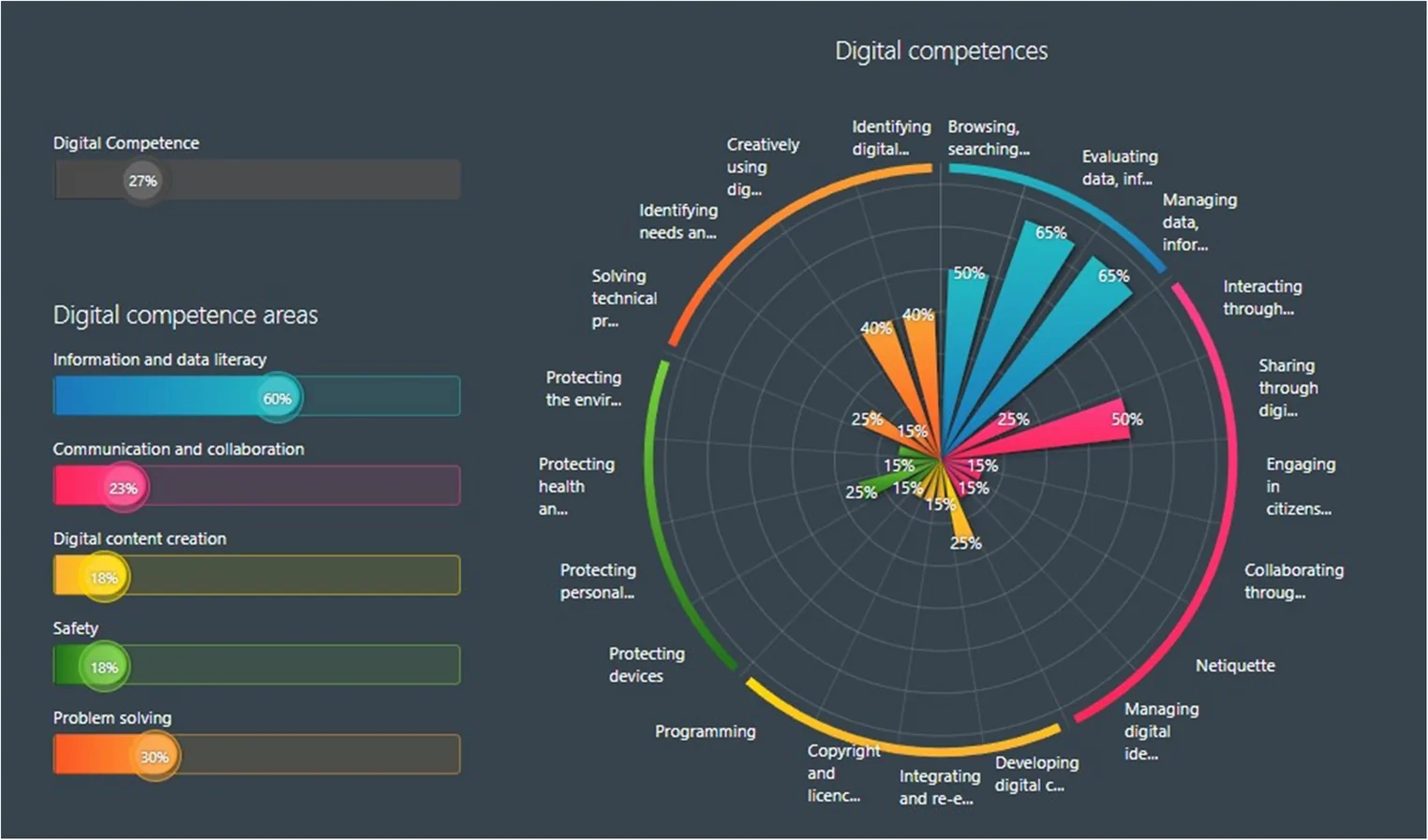
Mapping of DigComp 2.2 dimensions [14] identified in the IFBLS guidelines for core competence [7], which is the selected backward-looking source

**Fig. 5:**
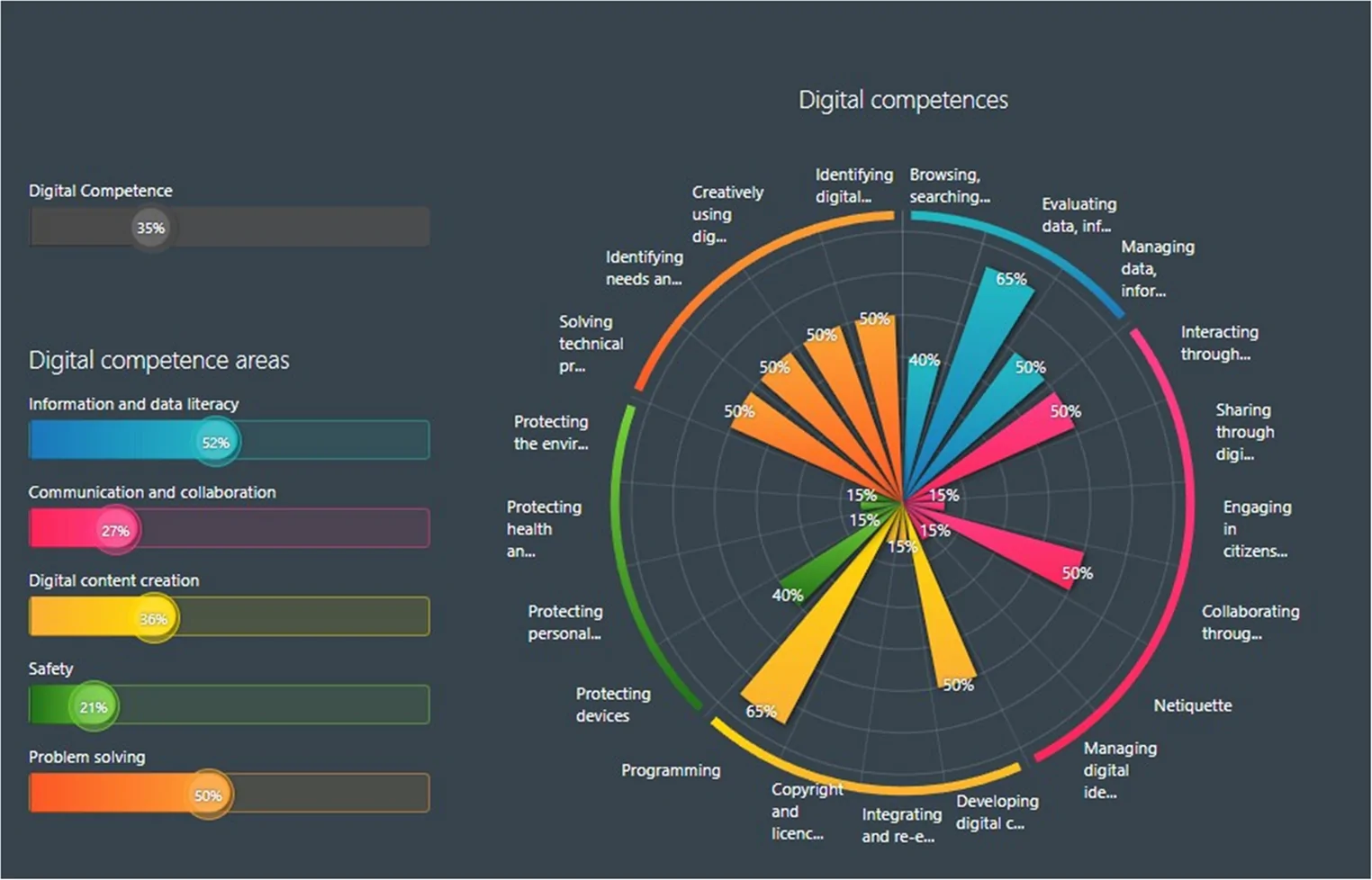
Mapping of DigComp 2.2 dimensions [14] identified in Adler et al. [27], which is the selected forward-looking source

To supplement the above analysis, we performed a quantitative analysis of one forward-looking (Fig. 4), and one backward-looking (Fig. 5) source based on the DigComp 2.2 framework. Here, we saw substantial differences in the distribution of competency dimensions between the forward- and the backward- looking perspectives, similar to the analysis using the Orlikowski & Gash framework.

The main difference is that in the forward-looking source [27] we detected a much broader representation of desired competencies than in the backward-looking source [7]; in the latter, by far the most “hits” were concentrated in the dimension *Information and data literacy*. In this dimension, the backward-looking source [7] focuses on traditional tasks like “*assess the validity of data/test results*”, while the forward-looking [27] source emphasizes advanced competences, such as “*big data and data analysis*”.

Within the dimension *digital content creation*, the forward-looking source [26], places notably stronger emphasis on the sub-competences *programming* and *developing digital content* such as “*experiences with simple programming language*” and “*programming and training of machine learning models*”. In addition, the dimension *problem solving* is more strongly represented in the forward-looking source [27], particularly in the sub-competence *Identifying needs and technological responses*, for example “*to understand and further develop the algorithms for data evaluation*”. Moreover, the desired competencies were distributed more evenly across all dimensions, with *Safety* being the one with fewest “hits”. Still, the forward-looking source [27] highlights a stronger focus on the sub-competence *protecting devices*,* for example “basics of IT security”.*

## Discussion and Perspectives

### What is TL in BLS?

One of the major findings in this paper is that the answer to this question depends on who and how you ask. In the initial screening of the sources, we discovered a dichotomy in whether the perspective on TL in the sources was mainly based on a backward- or a forward-looking perspective. This could be caused by the method or the focus of the sources. For example, some of the backward-looking sources [17, 20] were based on the Delphi method, which is a method for establishing consensus [32] and therefore can tend to condense around well-established areas. Others are guidelines and standards, which, by their nature, focus on established practices. In contrast, one of the forward-looking sources is an opinion paper that explicitly aims to argue for a focus on emerging technologies [29].

The next question is whether these different temporal perspectives reveal different answers to the question above. For the Orlikowski & Gash analysis the answer is yes, as differences emerge across the three domains of their framework in terms of what is emphasized in the forward-looking relative to the backward-looking perspectives.

This is most evident in the *Nature of Technolog*y domain, which is not very prevalent in the backward-looking sources, and is dominant in the forward-looking sources. Backward-looking sources tend to conceptualize technology in narrow, instrumental terms, focusing primarily on how specific devices operate; understanding technology is thus limited to grasping how instruments function [7, 22, 24, 26] or how digital systems like digital pathology [23] and LIS work in practice [19]. In addition, in several sources, this domain is either completely absent or only implicitly addressed [15–18, 20, 21, 25], reflecting a limited emphasis on deeper conceptual understanding of technology. In contrast, forward-looking sources emphasize the need to build a deeper foundational understanding of complex technologies, including computational thinking, AI, machine learning, data literacy, and digital pathology [2, 27–29]. This represents a major shift in both scope and depth of what it means to understand technology, as the forward-looking sources indicate that BLS will not only be expected to understand existing technologies, but to gain a more abstract understanding of the underlying principles of how technologies function.

Differences also appear in the two temporal perspectives in the domains *Technology Strategy* and *Technology in Use*. *Technology Strategy* is the least explicitly addressed domain in both the backward- and forward-looking sources. In backward-looking sources, it is rarely mentioned as a set of competencies. When mentioned, it tends to be embedded in practical concerns with a focus on restricting access to results [18, 20], optimizing workflow or improving efficiency [18, 19, 21, 26]. This suggests an implicit assumption that strategic thinking emerges from operational practice. Forward-looking sources demonstrate, albeit still largely implicitly, a more consistently focus on the purpose and value of technology [2, 27–29]. While not always framed as “strategy”, these sources highlight the need to understand the application of technologies like AI and robotics [2, 27, 28] and *why* technologies are introduced–for example, in ensuring quality and test optimization [2, 28]. While this domain is underrepresented in both temporal perspectives, forward-looking sources demonstrate a more intentional focus on *Technology Strategy.*

*Technology in Use* is the most consistently addressed domain across all sources, but it, too, is treated differently in the backward- and the forward-looking perspective. Backward-looking sources emphasize operational proficiency, focusing on the correct use of laboratory equipment, LIS systems, and instrument calibration, quality control, and basic digital tasks [7, 15, 16, 18–26]. These competencies are framed as clearly defined procedural tasks aligned with established workflows. In line with these findings, our own study of Danish BLS education [33] shows a similar application-oriented view of technology, where hands-on proficiency is prioritized. Forward-looking sources expand this scope as they include more advanced digital competencies such as programming, data analytics, machine learning and the operation of AI-supported diagnostics [2, 27–29]. In this way, *Technology in Use* within BLS, is not limited to execution of standardized procedures with a focus on compliance, but also encompasses interpretation, configuration, and troubleshooting in complex digital environments. Consequently, *Technology in use* expands from a task-oriented set of skills toward competencies that integrates critical thinking, problem-solving, and analytical evaluation.

The comparison of backward- and forward-looking perspectives in our analysis by Orlikowski & Gash´s framework thus reveals an ongoing shift both in how TL is understood within the BLS profession and in what is seen to be central to TL.

In the DigComp 2.2 analysis we likewise see differences between the temporal perspectives. The aspects of evaluating and managing data are dominant in the backward-looking sources, while digital content creation and problem solving are dominant in the forward-looking sources. Going into more detail we see that the forward-looking perspective of Adler et al. [27], has a strong emphasis on programming and solving technical problems. Thus, in both analyses, the backward- and forward-looking perspectives do indeed reveal different answers to the question: What is TL in BLS?

We used two different analytical frameworks, expecting that they might uncover different aspects of TL in BLS. And looking at what the two different analytical frameworks of Orlikowski & Gash and DigComp 2.2 reveal about TL in BLS we indeed detect differences in emphasis, while also finding many similarities. Both frameworks reveal a strong emphasis on evaluating and managing data. The aspect of operating and maintaining equipment, including new equipment, is mainly detected in the Orlikowski & Gash analysis and is not clear in the DigComp 2.2. framework. This could be because DigComp 2.2 is designed as a digital competence framework for all citizens, with a primary aim to capture broad skills across diverse contexts. Nevertheless, as Kluzer et al. [34] emphasize, DigComp´s adaptability has supported professional development across different sectors, underscoring its relevance in specialized areas such as BLS.

Comparing results of using these two different frameworks in the analysis thus leads us to speculate that an aspect that we might call *technical proficiency*, may be missing from the DigComp 2.2 framework when it is applied to the very specific context of BLS. This would be a competence dimension that goes beyond general digital literacy and refers to the profession-specific ability to operate, maintain, and handle both software and hardware of complex equipment. A similar argument is reflected in UNESCO´s *Digital Literacy Global Framework*, which emphasizes the need for the additional dimensions “0. Devices and software operations” and “6. Career-related competences” [35]. The first identifies technical and operational skills, and the latter highlights the ability to operate specialized digital technologies and handle specialized data and digital content within a particular field. Together, these dimensions acknowledge that TL in technology-intensive contexts such as BLS extends beyond general digital literacy and must encompass the ability to manage and interact with advanced domain-specific technologies.

The BLS profession is deeply rooted in, and strongly shaped by, technological and digital solutions and, as reflected in guidelines and studies on competency profiles, TL in BLS is currently primarily understood as the ability to master and understand laboratory technologies. This includes procedural proficiency, knowledge of quality standards, and the capacity to handle digital systems and basic data management. However, in the forward-looking sources, other aspects of TL are identified that will very likely, and very soon, become both central and important, particularly the need for foundational knowledge of complex technologies, including artificial intelligence, machine learning and digital pathology, as well as aspects of collaborating and interacting with and about digital technologies.

Going further, our analyses show that both forward- and backward-looking sources tend to underemphasize *Technological strategy*. This dimension goes beyond procedural proficiency and instead encompasses strategic and interpretative engagement with technology. Taken together, these points to an important gap: TL in BLS should not only involve the ability to operate laboratory technologies, from basic equipment to advanced systems, but also the ability to critically assess their purpose, and impact on workflow and diagnostic quality. In addition, it should include advanced operational skills such as configuration, troubleshooting, and data analysis, and the ability to engage with technology in a strategic-oriented way.

### Strengths and Limitations

Although we argue that it is a strength that the two models, which we have chosen to analyze the data, can uncover different aspects of technological literacy in the included sources, there will naturally also be elements that these two models do not find, and that might have been clearer if we had used other, or additional, models. A key strength of this study lies in the combination of two complementary analytical frameworks, enabling both a conceptual and a competency-based analysis of TL in BLS. It is also a strength in the analysis that we have been able to divide the sources into those, that describe existing, well-established practices (the “backward-looking”) and those, that explicitly take a forward-looking perspective. This allows us to describe both the existing landscape of technological literacy in BLS and also point to what is needed for BLS in the near future.

The heterogeneity of terminology across countries represents a potential limitation, as variations may have influenced the identification of relevant sources despite efforts to include a broad range of search terms. Furthermore, the small number of included sources reflects the emerging nature of TL within BLS, which may affect the generalizability of the findings, but underscores the need for further research.

### Insights from Other Health Professions

While this scoping review is, to the best of our knowledge, the first to systematically explore TL within BLS, other health professions, particularly nursing, physical therapy, and broader clinical education, have previously investigated similar topics.

In nursing education, a scoping review shows that integration of digital technologies such as electronic health records (EHR), simulations, and telehealth is often limited and lacks coherence across programs [36]. A study further notes the absence of structured pedagogical models that not only support the acquisition of technical skills, but also promote critical thinking, professional judgment, and ethical reflection in the use of digital tools [37]. Moreover, many nurse educators themselves often lack the digital competencies required to teach confidently and effectively in this domain [36].

In physical therapy, research has highlighted the importance of structured assessments of digital readiness, particularly in telehealth, where both practitioners and patients must navigate digital platforms confidently [38]. The American Physical Therapy Association has advocated for a “*digitally enabled physical therapist*” profile, with competencies in EHR, telepresence, wearables, and digital therapeutics [39], supported by educational interventions using virtual reality and dual-camera telepresence systems to enhance clinical accuracy and decision-making in remote care settings [39].

Across health professions education, approaches have been proposed to conceptualize TL, with a growing focus on artificial intelligence and data-driven competencies. Schubert et al. propose incorporating AI education into medical schools through a tiered framework of basic, proficient, and expert levels, tailored to clinicians’ roles and taught interdisciplinary [40]. Similarly, Doherty et al. identified a lack of structured AI training for radiographers and other imaging professionals, warning that this may hinder clinical integration [41]. Ittenbach proposes a clinical data science curriculum that integrates biostatistics and bioinformatics, thereby advancing data management from a technical service function to a recognized scientific discipline [42]. In pathology, Browning et al. likewise stress the need for structured training, as medical trainees welcomed the shift toward digital pathology but reported technical challenges, highlighting the need for combining digital and traditional microscopy [43].

A recent Delphi study across Europe, North America, and Asia identified four key domains of AI literacy for health professionals: foundational knowledge, practical application, experimental engagement, and ethical awareness [44].

### Implications for Education Programs

This work shows that TL in BLS encompasses both operational proficiency with laboratory equipment and digital skills, as well as an understanding of the underlying principles of laboratory technologies. Current competency profiles primarily focus on routine practices, including instrument operating, quality control, and data management, while perspectives on future competences highlight the importance of strategic thinking, data literacy, critical reflection and problem-solving, as well as competencies for new technologies such as AI and digital image analysis. As a result, graduates may currently have strong skills in routine technical tasks but lack competencies needed to engage with new technologies. To address this gap, BLS educational programs should aim to support digital and analytical competencies that foster more interpretative technological competences. Although complex technologies such as AI and machine learning are increasingly relevant, no studies were identified that clarify how these competencies should be introduced. We therefore suggest that further research should explore the appropriate scope and level at which foundational knowledge of such technologies should be introduced into curricula across different health educations. Based on the results of this review, curriculum development should explicitly integrate:

1. foundational understanding of emerging technologies such as artificial intelligence, data analytics, and digital pathology,
2. opportunities for students to engage in problem-solving and critical reflection on the use and implications of technology in clinical practice, and
3. structured collaboration with clinical training sites to ensure alignment with ongoing technological developments in laboratory settings.

These findings also suggest a need for learning environments that support collaborative engagement and exchange of knowledge in relation to technology, particularly in bridging the gap between operational competencies and more interpretative and strategically oriented forms of technological understanding.

Insights from studies on TL in relation to other health professions indicate that structured teaching approaches can support integration of these new competencies into the curriculum. An approach to this could be to support critical reflection and problem-solving competencies through pedagogical frameworks such as computational thinking, which can help students grasp how technologies function while also reflecting on limitations and potential [45]. In related health professions, including nursing and physical therapy, limited pedagogical coherence for integration of digital tools have been persistent challenges [37, 44]; here, also, a comprehensive framework, such as computational thinking, might help. Another approach could be simulation-based teaching, as used in nursing education, as this can effectively link theory and practice and allow students to experiment with technology and reflect on technological possibilities and limitations [36].

One of the sources in this review emphasizes the importance of strengthening collaboration with clinical education sites, where the technological development takes place [2]. Our own study on competencies of Danish BLS, also points to the need for closer integration between educational institutions and clinical practice for developing TL competency profiles [33]. Such collaboration could help clarify the appropriate scope and level for introducing complex technologies, and thereby guide formulation of learning goals, including taxonomic level, which again can guide teaching approaches. Embedding TL as a transversal learning objective, ideally co-developed with clinical partners, can support students to acquire technical proficiency while also develop the ability to critically evaluate the function and impact of technologies, participate in improving laboratory processes, and ensure their safe and effective use in practice [28, 43].

### Future Directions and Remaining Questions

While this review identifies overarching strategies and competencies for TL in BLS, it remains unclear how this should be operationalized and evaluated in educational practice. As educators, we still lack well-documented examples of approaches that foster TL in ways that mirror clinical reality and the rapid evolution of laboratory technologies. Future research should include experimental study designs to investigate how technological literacy develops and how specific educational interventions can support this development in authentic clinical contexts. Additionally, given the identified gap between operational use of technologies and a more critical understanding, there is a need to further investigate how such high-order competencies can be developed. Research could focus on learning designs that support collaborative engagement and exchange of knowledge around technology use. This may involve simulation-based training, or structured reflection embedded in clinical practice.

A central challenge is thus to determine how TL can be operationalized within the profession, at which levels in the curriculum it should be introduced, and how it can be assessed meaningfully. Simulation, computational thinking and collaboration with clinical practice appear as promising ways forward. However, more evidence is needed on how these can most effectively support students’ TL.

## References

[1] World Health Organization. Global strategy on digital health 2020–2025. Geneva: World Health Organization. 2021.

[2] Jensen CL, Thomsen LK, Zeuthen M, Johnsen S, El Jashi R, Nielsen MFB, et al. Biomedical laboratory scientists and technicians in digital pathology – Is there a need for professional development? Digit Health. 2024;10:20552076241237392. 38495864 10.1177/20552076241237392 PMC10943708

[3] Neumaier M. Diagnostics 4.0: the medical laboratory in digital health. Clin Chem Lab Med CCLM. 2019;57(3):343–8. 30530888 10.1515/cclm-2018-1088

[4] Khatab Z, Yousef GM. Disruptive innovations in the clinical laboratory: catching the wave of precision diagnostics. Crit Rev Clin Lab Sci. 2021;58(8):546–62. 34297653 10.1080/10408363.2021.1943302

[5] Babyar J. Laboratory science and a glimpse into the future. Int J Healthc Manag. 2020;13(sup1):456–63.

[6] ITEEA. Standards for Technological and Engineering Literacy (STEL) [Internet]. International Technology and Engineering Educators Association; 2022 [cited 2025 Apr 29]. Available from: https://www.iteea.org/downloadpurchase-stel

[7] The International Federation of Biomedical Laboratory Science. IFBLS Guidelines for Core Competence [Internet]. 2022 [cited 2024 Nov 23]. Available from: https://www.ifbls.org/index.php/statements/core-competence-core-curriculum

[8] Orlikowski WJ, Gash DC. Technological frames: making sense of information technology in organizations. ACM Trans Inf Syst. 1994;12(2):174–207.

[9] European Commission. DigComp 2.2, The Digital Competence framework for citizens: with new examples of knowledge, skills and attitudes. LU: Publications Office of the European Union; 2022.

[10] Shamseer L, Moher D, Clarke M, Ghersi D, Liberati A, Petticrew M, et al. Preferred reporting items for systematic review and meta-analysis protocols (PRISMA-P) 2015: elaboration and explanation. BMJ. 2015;349(jan02 1):g7647–7647. 10.1136/bmj.g7647 25555855

[11] Morgan RL, Whaley P, Thayer KA, Schünemann HJ. Identifying the PECO: a framework for formulating good questions to explore the association of environmental and other exposures with health outcomes. Environ Int. 2018;121:1027–31. 30166065 10.1016/j.envint.2018.07.015 PMC6908441

[12] Page MJ, McKenzie JE, Bossuyt PM, Boutron I, Hoffmann TC, Mulrow CD et al. The PRISMA 2020 statement: an updated guideline for reporting systematic reviews. BMJ. 2021;n71. 10.1136/bmj.n71 PMC8005924 33782057

[13] Eriksen MB, Frandsen TF. The impact of patient, intervention, comparison, outcome (PICO) as a search strategy tool on literature search quality: a systematic review. J Med Libr Assoc [Internet]. 2018 Oct 4 [cited 2023 Dec 31];106(4). Available from: http://jmla.pitt.edu/ojs/jmla/article/view/345 10.5195/jmla.2018.345 PMC6148624 30271283

[14] DigComp [Internet]. 2025 [cited 2025 Nov 3]. Available from: https://digcomp.digital-competence.eu/

[15] European Commission. Medical imaging and therapeutic equipment technicians [Internet]. 2024 [cited 2024 Jan 7]. Available from: https://esco.ec.europa.eu/en/classification/occupation?uri=http://data.europa.eu/esco/isco/C3212#overlayspin

[16] European Commission. Medical and pathology laboratory technicians [Internet]. 2024 [cited 2024 Jan 7]. Available from: https://esco.ec.europa.eu/en/classification/occupation_main#overlayspin

[17] Maekawa K, Kotera S, Ohsaki H. Competency for Japanese novice medical laboratory scientists: a Delphi method. BMC Med Educ. 2022;22(1):875. 36527049 10.1186/s12909-022-03878-7 PMC9756718

[18] Australian Institute of Medical and Clinical Scientists. Competency-based Standards for Medical Scientists [Internet]. 2022 [cited 2025 Feb 5]. Available from: https://aims.org.au/common/Uploaded%20files/Competency%20Based%20Standards/Competency-based%20Standards%20for%20MS_2022_Final.pdf

[19] Jimenez J, Alpaugh T. Using simulation to introduce students to a medical laboratory information system. Using Simul Introd Stud Med Lab Inf Syst. 2024;13(2):15–20.

[20] Stollenwerk MM, Gustafsson A, Edgren G, Gudmundsson P, Lindqvist M, Eriksson T. Core competencies for a biomedical laboratory scientist – a Delphi study. BMC Med Educ. 2022;22(1):476. 35725406 10.1186/s12909-022-03509-1 PMC9208704

[21] Millar BC, Tarasov A, Ternan N, Moore JE, Murphy C. Embedding Scientific Communication and Digital Capabilities in the Undergraduate Biomedical Science Curriculum. Br J Biomed Sci. 2023;80:11284. 37152115 10.3389/bjbs.2023.11284 PMC10154515

[22] Danmarks Evalueringsinstitut. Afdækning af teknologifokus i sundhedsuddannelserne: baggrundsrapport til projektet Teknologi i sundhedsprofessioner og -praksis. EVA; 2018.

[23] Mea VD, Carbone A, Di Loreto C, Bueno G, De Paoli P, García-Rojo M, et al. Teaching digital pathology: The International School of Digital Pathology and proposed syllabus. J Pathol Inform. 2017;8(1):27. 28828198 10.4103/jpi.jpi_17_17 PMC5545774

[24] Canadian Society for Medical Laboratory Science. CSMLS Competency Profile General Medical Laboratory Technologist. 2020.

[25] Institutet för biomedicinsk laboratorievetenskap. Kompetensbeskrivning för legitimerad biomedicinsk analytiker [Internet]. 2016 [cited 2025 Mar 10]. Available from: https://ibl-inst.se/om-yrket/kompetensbeskrivning/

[26] Health and Care Professions Council. Standards of proficiency for biomedical scientists [Internet]. 2023 [cited 2025 Mar 10]. Available from: https://www.hcpc-uk.org/standards/standards-of-proficiency/biomedical-scientists/

[27] Adler J, Lenski M, Tolios A, Taie SF, Sopic M, Rajdl D, et al. Digital competence in laboratory medicine. J Lab Med. 2023;47(4):143–8.

[28] Sharma A, Al-Haidose A, Al-Asmakh M, Abdallah AM. Integrating Artificial Intelligence into Biomedical Science Curricula: Advancing Healthcare Education. Clin Pract. 2024;14(4):1391–403. 39051306 10.3390/clinpract14040112 PMC11270210

[29] Jovičić SŽ, Vitkus D. Digital transformation towards the clinical laboratory of the future. Perspectives for the next decade. Clin Chem Lab Med CCLM. 2023;61(4):567–9. 36628420 10.1515/cclm-2023-0001

[30] Abidemi MM, Foloranmi OM, Opeyemi MM. A review on the trend of medical laboratory scientists training in Africa. Sokoto J Med Lab Sci. 2024;9(2):36–44.

[31] Kumar S, Chhabra G, Sehrawat KS, Singh M. Developing a competency assessment framework for medical laboratory technologists in primary healthcare settings in India. Horton S, editor. PLOS ONE. 2024;19(4):e0294939. 10.1371/journal.pone.0294939 PMC10984544 38557682

[32] Linstone HA, Turoff M. Delphi: a brief look backward and forward. Technol Forecast Soc Change. 2011;78(9):1712–9.

[33] Necip F, Lorenzen H, Qvist CC, Ellegaard M, Agustian HY. Technological literacy in biomedical laboratory science education: a mixed-methods study of curricula and student perspectives. BMC Med Educ. 2025;25(1):1124. 40731341 10.1186/s12909-025-07639-0 PMC12306041

[34] Kluzer S, Centeno C, O´Keeffe W. DigComp at work: the EU’s digital competence framework in action on the labour market: a selection of case studies. [Internet]. LU: Publications Office of the European Union; 2020 [cited 2025 Oct 11]. Available from: https://data.europa.eu/doi/

[35] Law N, Woo D, Torre J, de la, Wong G. A global framework of reference on digital literacy skills for indicator 4.4.2 - UNESCO Digital Library [Internet]. 2018 [cited 2025 Oct 11]. Available from: https://unesdoc.unesco.org/ark:/48223/pf0000265403

[36] Kleib M, Arnaert A, Nagle LM, Ali S, Idrees S, Costa DD, et al. Digital Health Education and Training for Undergraduate and Graduate Nursing Students: Scoping Review. JMIR Nurs. 2024;7:e58170. 39018092 10.2196/58170 PMC11292154

[37] Nes AAG, Steindal SA, Larsen MH, Heer HC, Lærum-Onsager E, Gjevjon ER. Technological literacy in nursing education: a scoping review. J Prof Nurs. 2021;37(2):320–34. 33867086 10.1016/j.profnurs.2021.01.008

[38] Rasekaba TM, Pereira P, Rani GV, Johnson R, McKechnie R, Blackberry I. Exploring telehealth readiness in a resource limited setting: digital and health literacy among older people in rural India (DAHLIA). Geriatrics. 2022;7(2):28. 35314600 10.3390/geriatrics7020028 PMC8938771

[39] APTA. The Digitally Enabled Physical Therapist. An APTA Foundational Paper [Internet]. 2022 [cited 2025 Aug 18]. Available from: https://www.apta.org/contentassets/e37aa1765cab4b1791d22717d3ac20af/apta-digital-health-foundational-paper-2022.pdf

[40] Schubert T, Oosterlinck T, Stevens RD, Maxwell PH, Van Der Schaar M. AI education for clinicians. eClinicalMedicine. 2025;79:102968. 39720600 10.1016/j.eclinm.2024.102968 PMC11667627

[41] Doherty G, McLaughlin L, Hughes C, McConnell J, Bond R, McFadden S. A scoping review of educational programmes on artificial intelligence (AI) available to medical imaging staff. Radiography. 2024;30(2):474–82. 38217933 10.1016/j.radi.2023.12.019

[42] Ittenbach RF. From clinical data management to clinical data science: time for a new educational model. Clin Transl Sci. 2023;16(8):1340–51. 37587756 10.1111/cts.13545 PMC10432862

[43] Browning L, Colling R, Rittscher J, Winter L, McEntyre N, Verrill C. Implementation of digital pathology into diagnostic practice: perceptions and opinions of histopathology trainees and implications for training. J Clin Pathol. 2020;73(4):223–7. 31597682 10.1136/jclinpath-2019-206137

[44] Beese AS, Jaks R, Alder E, Gani SMD. A Delphi study on health literacy competencies for health professionals. BMC Med Educ. 2025;25(1):38. 39789540 10.1186/s12909-024-06539-z PMC11715293

[45] Frank Jørgensen S, Heiberg T, Lorenzen H, Birk T, Møller E, Liikanen M. E, Case-study of teaching technological literacy for laboratory technological professions in the framework of computational thinking. Tidsskr Læring Og Medier LOM [Internet]. 2025 Aug 6 [cited 2025 Aug 27];18(32). Available from: https://tidsskrift.dk/lom/article/view/158611

